# Unraveling the pathogenic mechanism of a novel filamin a frameshift variant in periventricular nodular heterotopia

**DOI:** 10.3389/fphar.2024.1429177

**Published:** 2024-09-27

**Authors:** Chunran Xue, Yishu Wang, Jing Peng, Sisi Feng, Yangtai Guan, Yong Hao

**Affiliations:** ^1^ Department of Neurology, Renji Hospital, Shanghai Jiaotong University School of Medicine, Shanghai, China; ^2^ State Key Laboratory of Cell Biology, CAS Center for Excellence in Molecular Cell Science, Institute of Biochemistry and Cell Biology, University of Chinese Academy of Sciences, Chinese Academy of Sciences, Shanghai, China; ^3^ Department of Neurology, Punan Branch, Renji Hospital, Shanghai Jiaotong University School of Medicine, Shanghai, China

**Keywords:** filamin A, periventricular nodular heterotopia, epilepsy, induced pluripotent stem cell, f-actin

## Abstract

**Background:**

Periventricular nodular heterotopia (PVNH) is a neuronal migration disorder caused by the inability of neurons to move to the cortex. Patients with PVNH often experience epilepsy due to ectopic neuronal discharges. Most cases of PVNH are associated with variations in filamin A (*FLNA*), which encodes an actin-binding protein. However, the underlying pathological mechanisms remain unclear.

**Methods:**

Next-generation sequencing was performed to detect variants in the patient with PVNH, and the findings were confirmed using Sanger sequencing. Iterative threading assembly refinement was used to predict the structures of the variant proteins, and the search tool for the retrieval of interacting genes/proteins database was used to determine the interactions between FLNA and motility-related proteins. An induced pluripotent stem cell (iPSC) line was generated as a disease model by reprogramming human peripheral blood mononuclear cells. The FLNA expression in iPSCs was assessed using western blot and quantitative real-time polymerase chain reaction (qRT-PCR). Immunofluorescence analysis was performed to determine the arrangement of F-actin.

**Results:**

A novel *FLNA* frameshift variant (NM_001456.3: c.1466delG, p. G489Afs*9) was identified in a patient with PVNH and epilepsy. Bioinformatic analysis indicated that this variation was likely to impair FLNA function. Western blot and qRT-PCR analysis of iPSCs derived from the patient’s peripheral blood mononuclear cells revealed the absence of FLNA protein and mRNA. Immunofluorescence analysis suggested an irregular arrangement and disorganization of F-actin compared to that observed in healthy donors.

**Conclusion:**

Our findings indicate that the frameshift variant of *FLNA* (NM_001456.3: c.1466delG, p. G489Afs*9) impairs the arrangement and organization of F-actin, potentially influencing cell migration and causing PVNH.

## 1 Introduction

Periventricular nodular heterotopia (PVNH) is a neuronal migration disorder characterized by the inability of neurons to move from the periventricular region to the cortex during cortical formation (Sarkisian, et al., 2008; Sheen, et al., 2001), resulting in the abnormal distribution of neuronal nodules along the ventricles. Multiple genetic mutations are associated with PVNH, with filamin A (*FLNA*)-associated PVNH being the most common type (Sarkisian et al., 2008). Knockdown of *FLNA* in rats impairs neuronal migration, leading to increased susceptibility to seizures, highlighting the key role of *FLNA* in the pathogenesis of PVNH (Carabalona, et al., 2012).

The *FLNA* is located on the X chromosome and encodes an actin-binding protein. The relationship between *FLNA* and F-actin was demonstrated in 2001 using *FLN*A-deficient human melanoma cells (Flanagan, et al., 2001). These *FLNA*-deficient human melanoma cells exhibited different actin organization and were unable to maintain surface stabilization and support movement (Flanagan et al., 2001). Thus, it was hypothesized that cells without *FLNA* lose the ability to migrate from the lateral ventricle to the cerebral cortex because of the effect of the orthogonal actin network, and several studies have supported this deduction (Welter, et al., 2020; Hu, et al., 2017). Additionally, *FLNA*-deficient human seminoma cells exhibited irregular organization of F-actin and impaired cell motility (Welter et al., 2020). Another study demonstrated that the loss of *FLNA* in chondrocytes reduced F-actin fiber formation (Hu et al., 2017). However, the exact pathogenic mechanism underlying *FLNA*-associated PVNH and the relationship between *FLNA* and F-actin in PVNH remain unclear.

In addition to its role as an actin-binding protein, FLNA functions as a signaling protein by binding to numerous cellular components, including enzymes and channels, and modulating their activity (Nakamura, et al., 2011; van der Flier and Sonnenberg, 2001). For example, FLNA phosphorylation promotes golgi-to-lipid stripe rearrangement of Big two to activate Big 2-dependent adenosine diphosphate-ribosylation factor 1 at the cell membrane (Zhang, et al., 2013), which is required for cell migration and adhesion (Heuvingh, et al., 2007). Thus, the *FLNA* variant may also affect interactive proteins related to cell motility and further impair cell migration.

Over the past 2 decades, various *FLNA* variants have been identified in PVNH, including single-nucleotide variants, indels, and submicroscopic genomic copy number variations, with both familial and sporadic cases reported (Liu, et al., 2017; Parrini, et al., 2006). Patients with *FLNA*-associated PVNH often experience seizures (Sarkisian et al., 2008), though their intelligence and cognition are often normal (Lange, et al., 2015). Some individuals may present with extracerebral manifestations, including cardiovascular abnormalities, connective tissue disorders, and pulmonary hypoplasia (Gómez-Garre, et al., 2006; Lange et al., 2015; Masurel-Paulet, et al., 2011). Except for heterotopic nodules with periventricular distribution, magnetic resonance imaging (MRI) may reveal an enlarged cerebrospinal fluid space around the cerebellum, with normal cerebellar and fourth ventricle anatomy (Lange et al., 2015; Liu et al., 2017). Other brain malformations, such as corpus callosum hypoplasia and lateral deformation of the anterior horns, are also observed in some patients (Lange et al., 2015).

Herein, we report a novel frameshift variant in a patient with sporadic PVNH and epilepsy. Bioinformatics analysis revealed that this variant may be pathogenic. Peripheral blood mononuclear cells (PBMCs) were obtained from the patient and reprogrammed to generate an induced pluripotent stem cell (iPSC) line. Western blot (WB) and quantitative real-time polymerase chain reaction (qRT-PCR) results revealed the absent expression of FLNA in iPSCs derived from the patient. Furthermore, immunofluorescence analysis of iPSCs showed an irregular arrangement of F-actin in patient-derived iPSCs compared to that of healthy donor-derived iPSCs, providing an insight into the possible pathological mechanisms of *FLNA*-associated PVNH.

## 2 Materials and methods

### 2.1 Subjects

The patient was treated in the Department of Neurology, Ningbo Branch, Renji Hospital, Shanghai Jiao Tong University School of Medicine. The patient and her parents signed a consent form prior to participating in the study. The study was conducted in accordance with the Declaration of Helsinki and approved by the Medical Ethics Committee of Renji Hospital, Shanghai Jiao Tong University School of Medicine (KY 2022-074-B).

### 2.2 Genetic analysis

Next-generation sequencing (NGS) was performed to identify genetic variants. Briefly, DNA was isolated using Blood DNA Kit V2 (CW2553) following the manufacturer’s instructions and sheared. The KAPA Library Preparation Kit (Kapa Biosystems, KR0453) was used to prepare the DNA library. NGS analyses were conducted using the SeqCap Mixed Partitioning System. Sanger sequencing was performed to validate this variant. These outcomes match those of previous NGS analyses.

### 2.3 Protein structure modeling and constructing protein-protein interaction (PPI) network

The tertiary structure of the variant was predicted using the iterative threading assembly refinement (I-TASSER) software. PyMOL Molecular Graphics software was used to display the three-dimensional structure of the protein. The search tool for the retrieval of interacting genes/proteins (STRING) database was used to construct the PPIs for FLNA and visualized using Cytoscape software (version 3.8.2).

### 2.4 Generation and validation of the patient-derived iPSC line

Peripheral blood mononuclear cells were isolated using density centrifugation. The isolated and cultivated PMBCs were transduced with non-integrating episomal vectors according to the manufacturer’s protocol for the CytoTune-iPS 2.0 Sendai Reprogramming Kit (Thermo Fisher Scientific, Waltham, MA, United States). On Day 20, emergent stem cell colonies were manually selected, plated on Matrigel-coated plates, and multiplied in mTeSR1. When the cells reached 80% confluence, they were collected and seeded to evaluate their spontaneous differentiation. ADICON Clinical Laboratories Inc., Shanghai, China performed the karyotyping analysis.

### 2.5 Immunofluorescence staining

Cells were fixed in 4% paraformaldehyde for 15 min and incubated in phosphate-buffered saline (PBS) containing 5% bovine serum albumin (BSA) and 0.1% Triton X-100 for 30 min at room temperature. The cells were then treated with primary antibodies diluted in PBS containing 1% BSA overnight at 4°C, followed by an hour at room temperature with secondary antibodies. Cell nuclei were stained with 4′,6-diamidino-2-phenylindole (DAPI), and the cells were observed and photographed using a fluorescence microscope. The following antibodies were used: Pax6 (BD Biosciences), Brachyury (BD Biosciences, Franklin Lakes, NJ, United States), alpha-fetoprotein (AFP) (BD Biosciences), Phalloidin (APExBIO), and FLNA (Santa Cruz) were used for staining.

### 2.6 Flow cytometry

Single-cell suspensions were prepared, fixed, and permeabilized. Cells were stained for 30 min at 4°C with fluorochrome-conjugated antibodies, including phycoerythrin (PE) anti-stage-specific embryonic antigen-4 (SSEA-4), PE anti-octamer-binding transcription factor ¾ (OCT3/4), and PE anti-Nanog (BD Biosciences). Stained cells were analyzed using a FACSFortessa flow cytometer (BD Biosciences).

### 2.7 Western blot

The iPSCs were homogenized in lysis buffer containing protease inhibitors, placed on ice for 15 min, and centrifuged at 20,000 g for 15 min to isolate the protein supernatant. The supernatant was mixed with a 5-fold loading buffer, boiled for 10 min, resolved using sodium dodecyl sulfate-polyacrylamide gel electrophoresis on 10% gels (glyceraldehyde-3-phosphate dehydrogenase [GAPDH]) or 6% gels (FLNA) and blotted onto polyvinylidene fluoride membranes using a Mini-Trans-Blot system. The membranes were blocked with 5% BSA in tris-buffered saline Tween-20 for 1 h at room temperature and incubated with primary antibodies overnight at 4°C. The following day, the membranes were washed thrice, incubated with horseradish peroxidase (HRP)-conjugated secondary antibodies for 1 h at room temperature, and washed thrice. An enhanced chemiluminescence kit (Pierce, Apalachicola, KL, United States) was used to detect protein signals. The FLNA antibody (Santa Cruz, Dallas, TX, United States), GAPDH antibody (Proteintech, Rosemont, IL, United States), and HRP-conjugated mouse IgG antibody (Beyotime, Jiangsu, China) were used.

### 2.8 RNA extraction and qRT-PCR

RNA was extracted using an RNA kit from TIANGEN (Hebei, China) in accordance with the manufacturer’s instructions and reverse-transcribed using GoScript Reverse Transcriptase (Promega, Madison, WI, United States). The ABI Q6 (Life Technologies) system and SYBR Green Master Mix (Roche, Basel, Switzerland) were used to conduct qRT-PCR according to the manufacturer’s instructions. The primer sequences are listed in Table 1.

**TABLE 1:** Primer sequences used in qRT-PCR.

Gene	Primer	Sequence (5′to 3′)	Targeting exons
*FLNA*	Primer 1-F	GGA​AGA​AGA​TCC​AGC​AGA​ACA	Exon 3
	Primer 1-R	CCTCCAACAGCGCGATAA	Exon 3
*FLNA*	Primer 2-F	GGA​ACC​TGA​AGC​TGA​TCC​TG	Exon 3
	Primer 2-R	TCTTGGCCTCCTCATCCT	Exon 3
*FLNA*	Primer 3-F	GTG​ATC​AGC​CAG​TCG​GAA​AT	Exon 38
	Primer 3-R	TAA​ACT​CTG​CAG​GCT​CAA​AGG	Exon 38
*FLNA*	Primer 4-F	GCG​CTG​CTT​GTC​TTC​TTT​G	Exon 48
	Primer 4-R	CTT​TAT​TCC​TCT​TGG​CTG​GAG​A	Exon 48

## 3 Results

### 3.1 Clinical history

**FIGURE 1:**
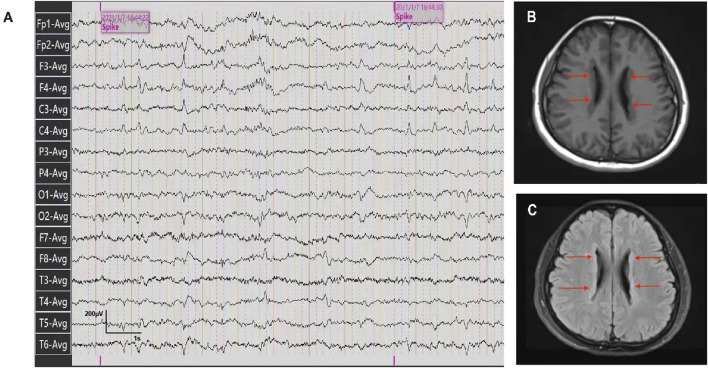
Ambulatory electroencephalogram and cranial MRI images of the patient. **(A)** Ambulatory electroencephalogram detected epileptiform discharges. **(B, C)** T1-weighted and T2 FLAIR images of the patient detected the bilateral periventricular nodular.

The patient was a 26-year-old Chinese female with a history of epilepsy. She experienced two seizures at intervals of a year. Before the seizures, the patient felt dazed and lost consciousness with blue lips and a pale complexion, but no limb twitching. The seizures continued for approximately 5–6 min resolving spontaneously. The patient didn’t exhibit developmental delay, cognitive impairment as well as psychiatric or behavioral abnormalities based on her statement and presentation. No perinatal injuries such as preterm labor or hypoxic-ischemic encephalopathy happened when she was born, but she experienced a febrile seizure at the age of one. No similar symptoms were observed in the patient’s relatives. The patient was diagnosed with epilepsy based on epileptiform discharges detected during an ambulatory electroencephalogram (Figure 1A). To further identify the etiology, cranial MRI was conducted and revealed multiple abnormal signals along the lateral ventricle bilaterally, which indicated PVNH (Figures 1B, C). No obvious abnormalities were observed on Holter monitoring or cardiac ultrasonography.

### 3.2 Identification of a novel frameshift variant of *FLNA*

**FIGURE 2:**
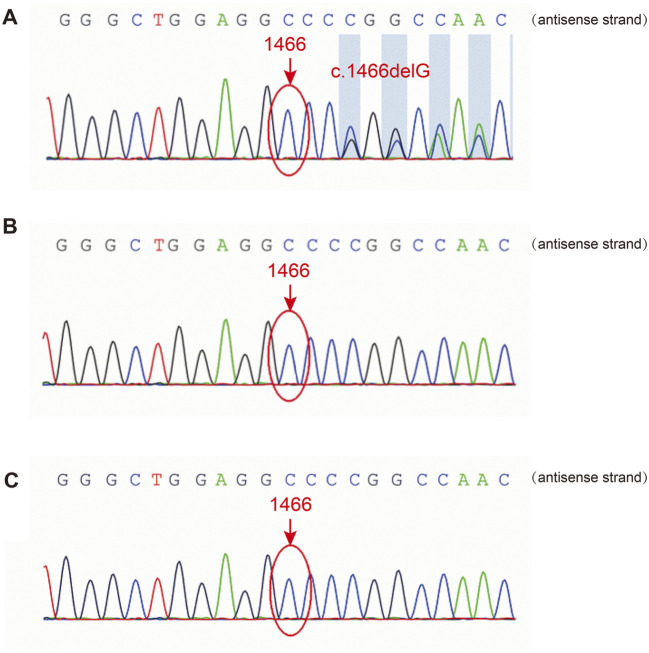
Sanger sequencing of *FLNA* in the patient and her parents. **(A)** The *FLNA* variant (c.1466del, p. G489Afs*9) was detected in the patient. **(B, C)** The variant was not detected in her father or mother.

A heterozygous frameshift variant, c.1466delG, in *FLNA* was identified in the patient’s PBMCs using NGS, leading to changes in the protein sequence p. G489Afs*9. Sanger sequencing confirmed the presence of this variant (Figure 2A), while it was absent in *FLNA* of her biological parents (Figures 2B, C). Thus, it was clearly a sporadic variant that has not been reported previously in the existing databases (PubMed, HGMDpro, gnomAD, and ClinVar). Additionally, the variation was rated as “pathogenic” according to the American College of Medical Genetics and Genomics guidelines.

### 3.3 Structure analysis suggests loss of function in the variant FLNA

**FIGURE 3:**
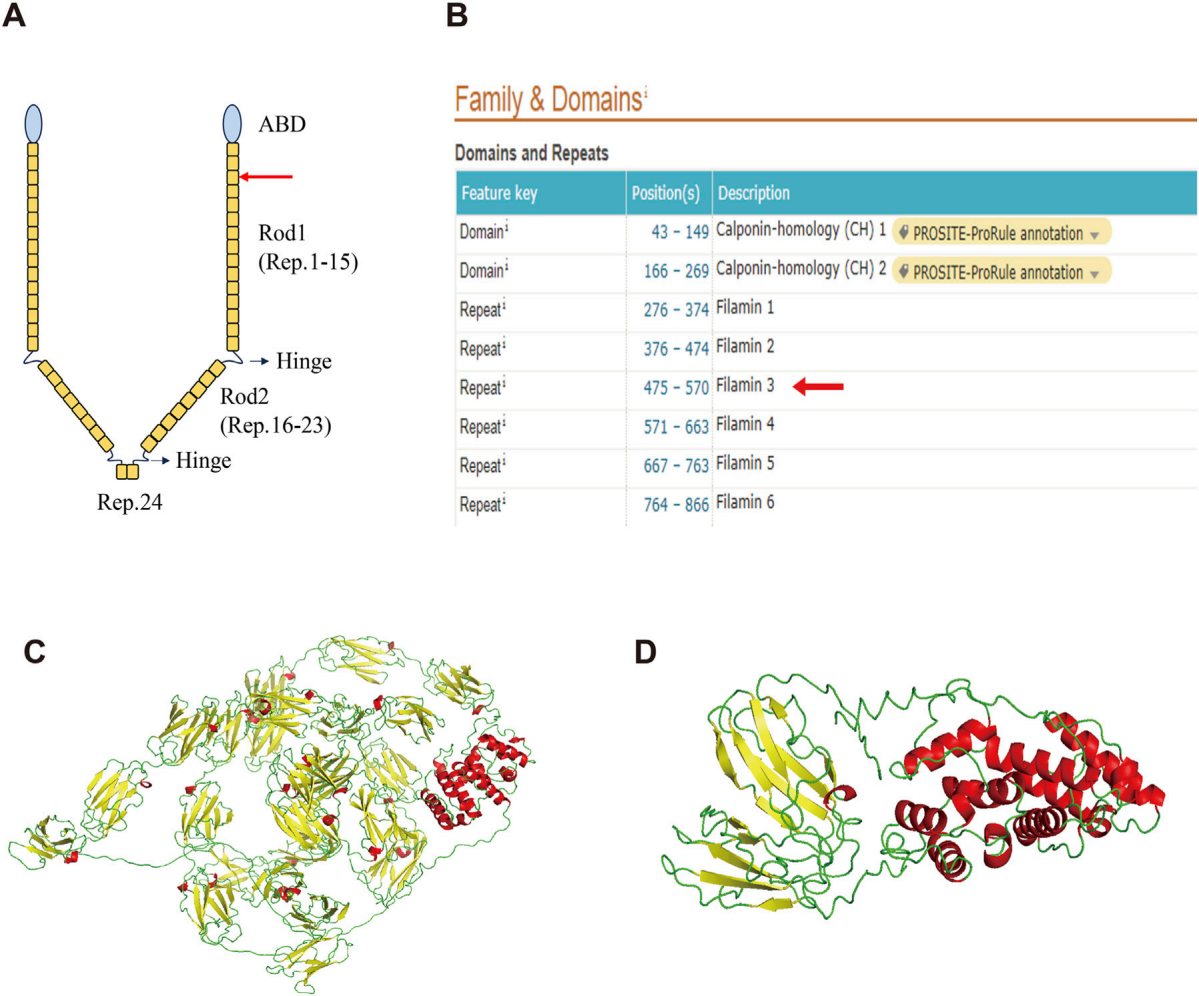
Structure of the wild-type FLNA and variant FLNA. **(A)** Schematic diagram illustrates the structure of FLNA. The red arrow indicates the variant and termination sites. **(B)** Domain positions of FLNA as shown in the UniProt database. Both the variant and termination sites are located in Rep. 3 (red arrow). **(C)** Predicted overall structure of wild-type FLNA from the AlphaFold Protein Structure database. **(D)** Predicted structure of the variant FLNA using I-TASSER. α-helix is shown in red, β-sheet in yellow, and random coil in green.

FLNA consists of one conserved actin-binding domain (ABD) and 24 repeat immunoglobulin-like domains (Zhou, et al., 2021) (Figure 3A). Two calpain-sensitive hinges segment the 24 domains into Rod1 (Reps. 1–15), Rod2 (Reps. 16–23), and Rep.24 (Zhou et al., 2021). The ABD domain, which contains two calponin homology sequences, facilitates actin binding (van der Flier and Sonnenberg, 2001). Rod1 is an extended chain, while Rod2 is more flexible and highly variable with a folded structure (Zhou et al., 2021). Rep.24 mediates the dimerization of FLNA (Nakamura, et al., 2007).

The deletion of this nucleotide results in a frameshift variant of *FLNA*, altering the 489th amino acid from glycine to alanine and introducing a premature stop codon. This results in the early termination of protein synthesis, leading to truncated protein length, from 2647 to 496 amino acids, significantly affecting structure and function. Based on the UniProt database, both the variant and termination sites of the protein were located in Rep. 3 (Figure 3B). The predicted overall structure of the wild-type FLNA in the AlphaFold Protein Structure Database is presented in Figure 3C, and the structure of the variant protein was predicted using I-TASSER (Figure 3D). Parts of Rod 1, Rod 2, and Rep. 24 are absent in the putative variant protein structure. The FLNA exists mainly as a homodimer that binds to F-actin, which is mediated by Rep. 24, as depicted in Figure 3A. Its function in binding to actin and affecting orthogonal branching relies on the formation of a homodimer, while the monomer does not function (Zhou et al., 2021). Therefore, the variant protein is likely to lose its original function due to structural changes.

### 3.4 FLNA is related to proteins involved in cell migration

**FIGURE 4:**
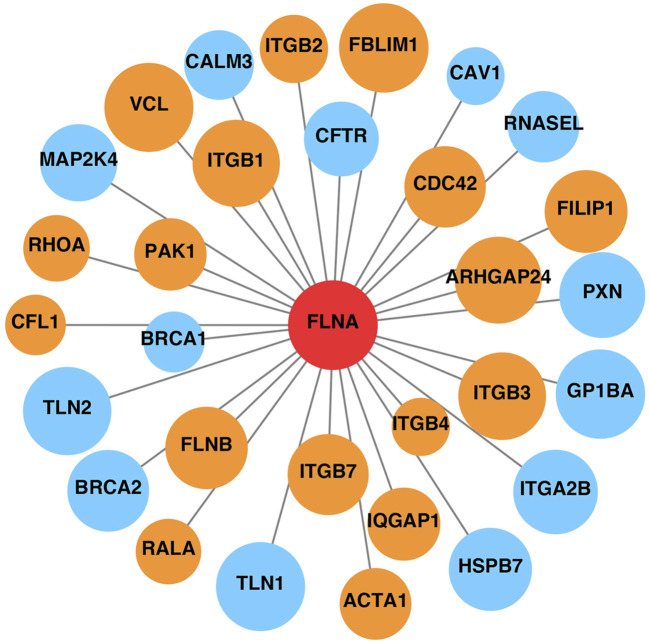
Protein-protein interaction network of FLNA. Proteins related to cell migration are marked in orange, and other proteins unrelated to cell migration are marked in blue. The area of the circles is proportional to the combined score between FLNA and related proteins.

Additionally, FLNA serves as a scaffold for signaling proteins, many of which are involved in cell movement and migration. The STRING database was used to construct the PPI network of FLNA (Figure 4). Proteins that interacted directly with FLNA were retained in the network. Based on the functions recorded in the UniProt database, proteins related to cell migration are marked in orange, such as CDC42 and the integrin ITGB2. Therefore, the PPI network indicated that FLNA plays an important role in cell migration as a signaling protein. The loss of FLNA function is likely to affect cell migration by affecting cell signaling.

### 3.5 Successful generation of patient-derived iPSC line

**FIGURE 5:**
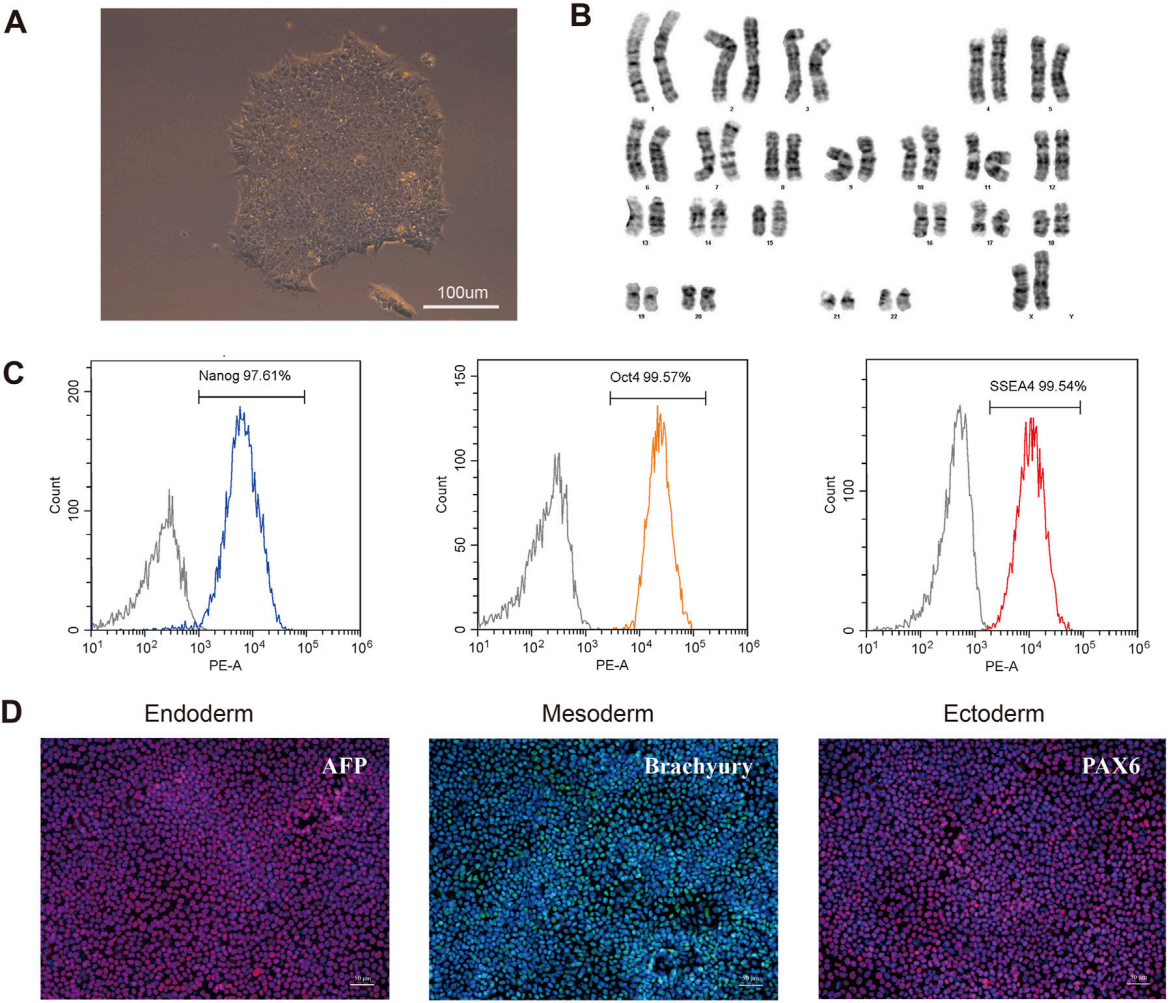
Characterization of the patient-derived iPSC. **(A)** Morphology of selected iPSC colonies. Scale bar: 100 µm. **(B)** Karyotype analysis of the iPSC line indicated no detectable abnormality. **(C)** Expression of pluripotent markers was confirmed by flow cytometry analysis (Nanog, OCT4, SSEA4). **(D)** Differentiation capacity of iPSCs into the three germ layers: ectoderm (Pax6), mesoderm (Brachyury) and endoderm (AFP) markers, demonstrated by immunofluorescent staining. Scale bar: 50 µm.

To further investigate the pathogenesis of the *FLNA* variant that causes PVNH and epilepsy, an iPSC line was generated from the PBMCs of patients. The PBMCs were transduced with the non-integrating reprogramming factors OCT3/4, KLF, c-Myc, and Sox 2, and iPSCs were selected after 20 days of transduction (Figure 5A). Karyotyping analysis did not detect any abnormalities and revealed a diploid 46 and XX karyotype (Figure 5B). Expression of the pluripotency markers Nanog, SSEA4, and OCT4 was confirmed using flow cytometry (Figure 5C). The ability of iPSCs to differentiate into the three germ layers was characterized by the expression of Pax 6 (ectoderm), Brachury (mesoderm), and AFP (endoderm) (Figure 5D). Therefore, these results indicated that the iPSC line was successfully generated.

### 3.6 *FLNA* variant impairs F-actin arrangement

**FIGURE 6:**
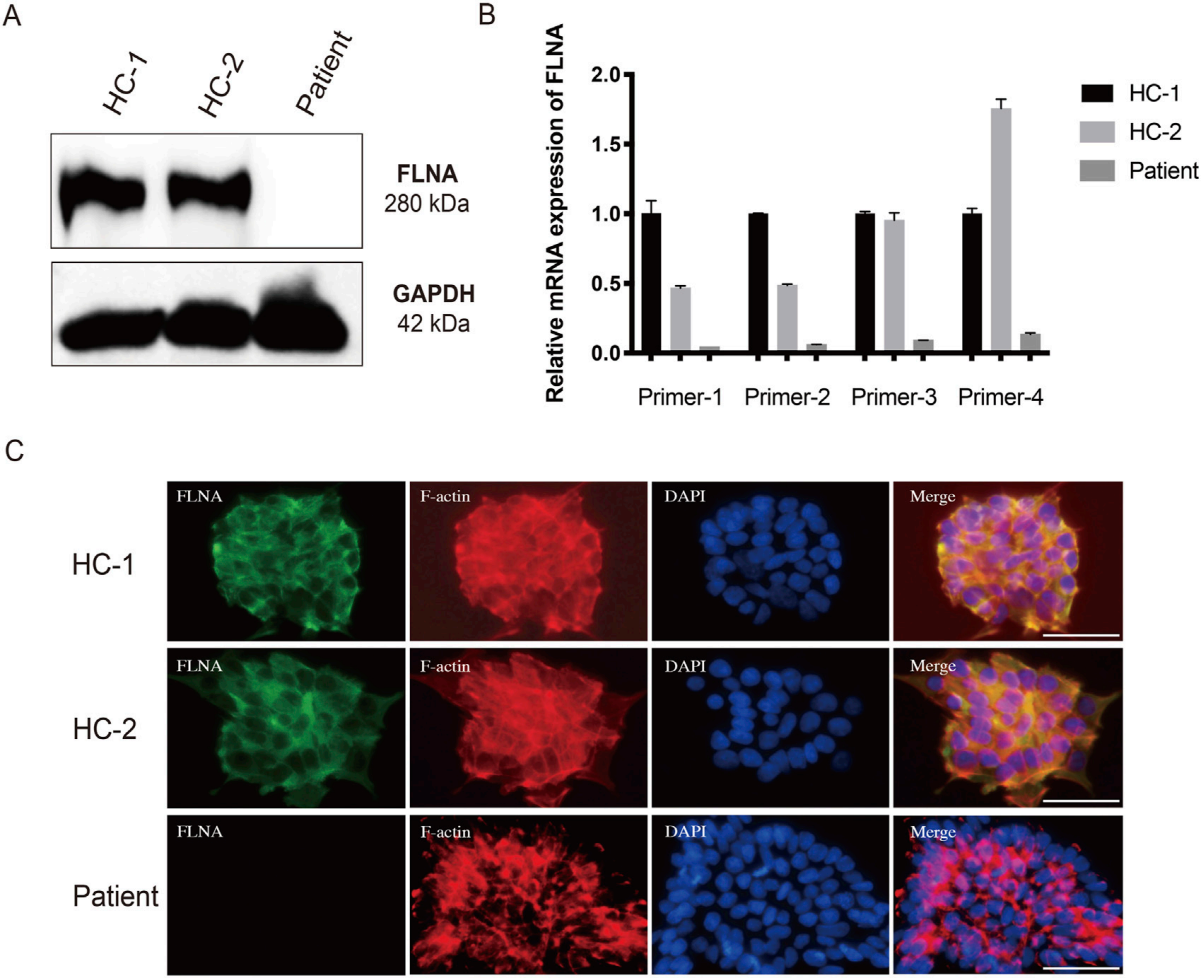
Loss of FLNA impairs the arrangement of F-actin in patient-derived iPSCs. **(A)** Protein levels of FLNA was tested using western blot. GAPDH served as internal reference. **(B)** The mRNA expression of wild-type and variant FLNA was assessed using quantitative polymerase chain reaction analysis. **(C)** Arrangement of F-actin illustrated using immunofluorescence. iPSCs stained with immunolabeled antibodies for FLNA (green) and phalloidin for F-actin (red). Nuclei (blue) were visualized using DAPI. Merged images are shown on the right. Scale bar: 25 µm.

To further investigate the expression of *FLNA* in the patient, WB and qRT-PCR were performed using patient-derived iPSCs. Notably, WB revealed a complete absence of wild-type protein expression in patient-derived iPSCs compared to iPSCs generated from two healthy donors (Figure 6A). Four primers targeting different loci of the wild-type *FLNA* gene were designed to detect mRNA levels of wild-type *FLNA*. The FLNA variant site of *FLNA* is located in exon 10. Primers 1 and 2 were designed to be complementary to the sequences in exon 3, whereas primers 3 and 4 were designed to be complementary to the sequences in exons 38 and 48, respectively. Thus, the mRNA of the wild-type and variant *FLNA* can be detected by primers 1 and 2, whereas only the mRNA of wild-type *FLNA* can be detected by primers 3 and 4. The results suggested that none of the four primers detected mRNA of variant *FLNA* (Figure 6B), indicating that the variant *FLNA* may not be transcribed or degraded immediately after transcription.

As an actin-binding protein, FLNA has been implicated in the promotion of the orthogonal branching of the actin network, thereby impacting cell migration. Consistent with the WB results, immunofluorescence staining of wild-type FLNA revealed the absence of FLNA in patient-derived iPSCs (Figure 6C). Additionally, immunofluorescence staining revealed that F-actin was more neatly arranged in healthy donor-derived iPSCs, with higher expression in the pericellular area near the cell membrane. However, F-actin was more irregularly arranged and disorganized in patient-derived iPSCs (Figure 6C). Moreover, the F-actin arranged along the cell periphery appeared to be broken. Disorders of the F-actin network may be responsible for impaired cell migration.

## 4 Discussion

PVNH is a malformation of the cerebral cortex characterized by heterotopic neurons lining the lateral ventricles owing to the failed migration of neurons during cortical formation (Sarkisian et al., 2008). Affected patients may present with epilepsy due to ectopic discharge. The *FLNA* is one of the identified genes associated with PVNH(Sarkisian et al., 2008). Multiple *FLNA* variants have been reported in patients with PVNH. In our study, we identified a novel *FLNA* frameshift variant (NM_001456.3: c.1466delG, p. G489Afs*9) in a patient with PVNH with epilepsy as a sporadic case, which has not been reported in the available databases (PubMed, HGMDpro, gnomAD, and ClinVar).

*FLNA* is located on the X-chromosome and encodes a 280 kDa protein comprising an ABD domain and 24 repeat β-sheets resembling the immunoglobulin domain (Zhou et al., 2021). The ABD of *FLNA* allows the protein to bind to F-actin and is involved in cytoskeletal organization (Yamazaki, et al., 2002). The newly discovered *FLNA* variant (NM_001456.3: c.1466delG, p. G489Afs*9) leads to the premature introduction of a stop codon, resulting in the shortening of the protein length from 2647 to 496 amino acids, so the variant protein is composed of only the ABD domain, Rep 1-2, and incomplete Rep3. Massive loss of bases may cause nonsense-mediated mRNA decay, an important mechanism that detects premature termination codons and triggers mRNA degradation to avoid the accumulation of truncated and potentially harmful proteins. Additionally, if the mRNA remains and is transcribed into a protein, the large number of lost fragments significantly alters the structure of FLNA based on the prediction, and furthermore, it is highly likely to affect the function of the protein.

In addition to the ABD domain, the Rep1-24 domain plays an essential role and serves as a scaffold for various binding partners, including receptors, channels, and intracellular signaling molecules (Zhou et al., 2021). Many FLNA-partner complexes are involved in the regulation of cell adhesion and motility. The PPI network of FLNA showed its interactions with a wide range of proteins, many of which are associated with cell adhesion, spreading, and migration. For example, CDC42 is a small GTPase belonging to the Rho family, which serves as a key regulator of actin dynamics. CDC42 plays an important role in inducing actin reorganization and filopodium formation, thus regulating cell migration (Nobes and Hall, 1995; Ohta, et al., 1999).

We successfully generated iPSCs from the patients’ PBMCs, which were used to further explore pathogenic mechanisms. Results of WB and qRT-PCR revealed the absence of both wild-type and variant *FLNA* in iPSCs derived from the patient, which was unexpected. The variant in our study was heterozygous on the X chromosome. According to the X chromosome stochastic inactivation theory, random X chromosome inactivation shuts down gene transcription on one of the two X chromosomes in females (Harper, 2011). Thus, some of the cells in the patient were expected to express wild-type *FLNA*. Many factors affect cellular transcription, including epigenetic and transcription factor regulation. The skewing of X chromosome inactivation may be responsible for this result. An unusual degree of skewing of X-chromosome inactivation was identified in females with X-linked gene mutations even in a female without any X-chromosomal abnormality; however, the reason for this remains unknown (Tukiainen, et al., 2017; Viggiano, et al., 2016). Whether skewed inactivation occurred in this patient requires further investigation.

Although an association between *FLNA* mutations and PVNH has been demonstrated, the mechanism by which *FLNA* defects cause PVNH remains controversial. Several studies have demonstrated that loss of FLNA disrupts the arrangement of the F-actin cytoskeleton and impairs cell motility in tumor cells (Flanagan et al., 2001; Welter et al., 2020). However, whether the *FLNA* variants affect the F-actin cytoskeleton in PVNH remains unknown. In this study, we investigated the F-actin network in iPSCs derived from patients with PVNH and two healthy donors. F-actin staining showed an irregular arrangement and organization of the F-actin network in patient-derived iPSCs compared to that of healthy donor-derived iPSCs. F-actin in iPSCs from the two healthy donors was neatly organized in the cytoplasm, whereas in iPSCs from patients, it appeared to be broken and irregularly arranged. These results suggested that FLNA is essential for regulating the orthogonal structure of F-actin. Therefore, the loss of FLNA function may impair cell migration by influencing the arrangement of F-actin, resulting in the migration failure of newborn neurons and PVNH. To our knowledge, this is the first study exploring the pathogenesis of *FLNA*-associated PVNH in patient-derived iPSCs. Our results are similar to previous findings that FLNA deficiency impaired cell motility by affecting the arrangement of F-actin in human seminoma cells and melanoma cells (Flanagan et al., 2001; Welter et al., 2020). Several studies have explored the mechanisms underlying PVNH in animal models. Carabalona et al. found that *FLNA*-knockdown rats exhibited PVNH. They did not focus on the arrangement of F-actin but suggested that disorganization of radial glia may be the cause of *FLNA*-associated PVNH(Carabalona et al., 2012). Whether the same phenomenon occurs in animal models warrants further exploration. However, it is reported that *FLNA*-deficient mice showed no differences in F-actin levels and didn’t exhibit PVNH (Feng, et al., 2006; Hart, et al., 2006). There are several possible explanations for the findings. Both teams studied fibroblasts, which are inherently less motile, and therefore may not cause significant differences in F-actin and cell motility. In addition, it is possible that in the neonatal neuronal cells of mice, FLNB, another actin-binding protein, strongly compensates for the function of FLNA; therefore, no significant ectopic neuronal nodules appear.

Here, we report a novel variant of *FLNA* in a patient with epilepsy and PVNH. Although variants of *FLNA* have been found to cause PVNH, the underlying pathogenic mechanism remains unclear. We successfully generated an iPSC line from the patient’s PBMCs and discovered that the variant caused the absence of FLNA, which impaired the arrangement of F-actin. Our findings suggest that this variant of *FLNA* plays a role in cell migration and the pathogenesis of *FLNA*-associated PVNH. To date, no study has used cells from patients to investigate this mechanism, making our findings important. However, this study has some limitations. As the results were based on a single patient, a larger population of patients is needed to further identify the mechanism. Additionally, the migration ability was not evaluated, and it was necessary to differentiate iPSCs into neurons *in vitro* to further study their migration and discharge functions. Moreover, the mechanism of epilepsy in the *FLNA*-associated PVNH is still unclear. Several clinical studies have found that heterotopic lesions produce aberrant connectivity with normal brain regions and both of them are involved in the development of epileptogenic networks (Jacobs, et al., 2009; Pizzo, et al., 2017), but the exact mechanism needs to be explored further in an animal model of *FLNA*-associated PVNH.

## Data Availability

The original contributions presented in the study are publicly available. This data can be found here: https://www.ncbi.nlm.nih.gov/sra/PRJNA1163365.
