# Supplementary material for: Unraveling the pathogenic mechanism of a novel filamin a frameshift variant in periventricular nodular heterotopia
Source: Front Pharmacol. 2024 Sep 27;15:1429177. doi: 10.3389/fphar.2024.1429177 (PMC11466872; doi:10.3389/fphar.2024.1429177)
Supplement: Supplementary file 1 [file Table1.docx]

The ten threading templates used by I-TASSER and their normalized Z-score.

| **Rank** | **PDB Hit** | **Iden1** | **Iden2** | **Cov** | **Norm. Z-score** |
| --- | --- | --- | --- | --- | --- |
| 1 | 3ferA | 0.88 | 0.42 | 0.47 | 1.89 |
| 2 | 2k7pA | 0.24 | 0.13 | 0.38 | 1.80 |
| 3 | 2wa7A | 0.88 | 0.40 | 0.45 | 3.85 |
| 4 | 4b7lA | 0.85 | 0.56 | 0.66 | 1.98 |
| 5 | 4b7lA | 0.72 | 0.56 | 0.67 | 3.97 |
| 6 | 4b7l | 0.85 | 0.56 | 0.66 | 2.67 |
| 7 | 4b7l | 0.72 | 0.56 | 0.66 | 1.96 |
| 8 | 4b7lA | 0.73 | 0.56 | 0.66 | 3.70 |
| 9 | 4b7l | 0.85 | 0.56 | 0.66 | 3.14 |
| 10 | 4b7lA | 0.84 | 0.56 | 0.66 | 1.91 |
